# MutLα Heterodimers Modify the Molecular Phenotype of Friedreich Ataxia

**DOI:** 10.1371/journal.pone.0100523

**Published:** 2014-06-27

**Authors:** Vahid Ezzatizadeh, Chiranjeevi Sandi, Madhavi Sandi, Sara Anjomani-Virmouni, Sahar Al-Mahdawi, Mark A. Pook

**Affiliations:** Division of Biosciences, School of Health Sciences and Social Care, Brunel University London, Uxbridge, United Kingdom; Alexander Fleming Biomedical Sciences Research Center, Greece

## Abstract

**Background:**

Friedreich ataxia (FRDA), the most common autosomal recessive ataxia disorder, is caused by a dynamic GAA repeat expansion mutation within intron 1 of *FXN* gene, resulting in down-regulation of frataxin expression. Studies of cell and mouse models have revealed a role for the mismatch repair (MMR) MutS-heterodimer complexes and the PMS2 component of the MutLα complex in the dynamics of intergenerational and somatic GAA repeat expansions: MSH2, MSH3 and MSH6 promote GAA repeat expansions, while PMS2 inhibits GAA repeat expansions.

**Methodology/Principal Findings:**

To determine the potential role of the other component of the MutLα complex, MLH1, in GAA repeat instability in FRDA, we have analyzed intergenerational and somatic GAA repeat expansions from *FXN* transgenic mice that have been crossed with Mlh1 deficient mice. We find that loss of Mlh1 activity reduces both intergenerational and somatic GAA repeat expansions. However, we also find that loss of either Mlh1 or Pms2 reduces *FXN* transcription, suggesting different mechanisms of action for Mlh1 and Pms2 on GAA repeat expansion dynamics and regulation of *FXN* transcription.

**Conclusions/Significance:**

Both MutLα components, PMS2 and MLH1, have now been shown to modify the molecular phenotype of FRDA. We propose that upregulation of MLH1 or PMS2 could be potential FRDA therapeutic approaches to increase *FXN* transcription.

## Introduction

Friedreich ataxia (FRDA) is a fatal, autosomal recessive neurodegenerative disorder caused by homozygous GAA repeat expansion within intron 1 of the *FXN* gene [1]. This mutation induces heterochromatin formation [2], likely due to abnormal non-B DNA or DNA**•**RNA hybrid triplex structures [3], [4], leading to *FXN* gene silencing and thus reduced expression of the essential mitochondrial protein, frataxin [5]. Frataxin insufficiency culminates in mitochondrial iron accumulation and reduced activity of iron-sulfur (Fe-S) cluster enzymes, including mitochondrial respiratory chain complexes and aconitase [6], leading to increased susceptibility to oxidative stress and resultant cell degeneration. The primary sites of FRDA pathology are the large sensory neurons of the dorsal root ganglia (DRG) and the dentate nucleus of the cerebellum [7]. However, there are also non-neuronal tissue dysfunctions including diabetes and cardiomyopathy, followed by death commonly in early adulthood [8], [9]. Thus far, there is no effective therapy for FRDA. Unaffected individuals carry *FXN* alleles containing 5–32 GAA repeats, while this is expanded to approximately 70–1700 GAA repeats in affected individuals, most commonly between 600–900 repeats. The GAA repeats are dynamic in FRDA patients, presenting both somatic and intergenerational GAA repeat instability [10], [11]. Somatic GAA repeat expansion takes place progressively in many different tissues throughout life, particularly in the DRG and cerebellum [12], [13]. In addition, different modes of intergenerational repeat instability have been detected. Thus, during maternal transmissions of GAA repeat expansions, further expansions and contractions are equally detected, while a bias towards contraction is observed during paternal transmissions [14]–[17]. These findings indicate that GAA repeat expansion dynamics might perform a critical role in FRDA disease progression, and hence finding approaches to prevent GAA repeat expansions or induce repeat contractions could be an effective strategy to treat this disorder. To study *FXN* GAA repeat instability and pathogenesis in FRDA, we have previously established two lines of human FRDA YAC transgenic mice, YG8 and YG22, containing GAA repeat expansion mutations within the *FXN* transgene [18]. When crossed onto a mouse *Fxn*-null genotype, these transgenic mice act as suitable models to study the molecular mechanisms of *FXN* dysfunction and to investigate FRDA therapeutic strategies [19]–[24]. For instance, investigation of YG8 and YG22 transgenic mice has revealed the age dependence and tissue selectivity of somatic GAA repeat expansions, particularly in cerebellum and DRG tissues [18], [25].

Previous investigations of mouse models have indicated the role of some mismatch repair (MMR) proteins in the CAG and CTG repeat instability dynamics of the other trinucleotide repeat (TNR) disorders, such as Huntington disease (HD) and myotonic dystrophy type 1 (DM1) respectively [26]. By analyzing intergenerational transmissions of YG8 and YG22 mouse models, we have similarly demonstrated that deficit of any of the Msh2, Msh3, Msh6 or Pms2 parental MMR proteins increases GAA repeat mutability (expansion and/or contraction) in the offspring. Subsequently, we have shown that loss of MMR-MutSα heterodimer protein components, Msh2 or Msh6, leads to a significant decline in somatic GAA repeat expansions. In contrast, loss of Pms2 protein increases GAA repeat expansions in neuronal tissues, particularly the cerebellum and DRG. However, this effect is not detectable in non-neuronal tissues, which are less susceptible to *FXN* GAA repeat instability [27].

Mechanistically, MutS-heterodimers are recruited to recognize base-base mismatches or small nucleotide insertion/deletion loops (IDLs) during MMR function. This procedure is then continued within eukaryotes by another MMR heterodimer complex, named MutL. This heterodimer complex comprises 4 different homologues: MLH1, MLH3, PMS1 and PMS2. MLH1 plays a central role by interacting with PMS2, PMS1 or MLH3 to form the three heterodimeric complexes MutLα, MutLβ and MutLγ, respectively. MutLα-heterodimers can interact with both MutSα and MutSβ, but MutLγ is only able to interact with MutSβ during the MMR process. The precise function of MutLβ is not yet determined [28], [29]. Although several studies have revealed a crucial role for MSH2, MSH3, MSH6 and PMS2 proteins on the dynamics of trinucleotide repeat based diseases, there are limited investigations of the MLH1 protein. However, recent studies of Huntington disease *Hdh^Q111^* transgenic mice have shown that Mlh1 is required to increase the CAG repeat expansion in somatic tissues [30]. To study the potential role of Mlh1 protein on intergenerational and somatic GAA repeat instability, we have crossed YG22 transgenic mice with mice that are deficient for Mlh1. Our findings demonstrate that Mlh1 promotes GAA repeat expansions within intergenerational transmissions and within selective somatic tissues. Moreover, to explore the effect of Pms2 or Mlh1 function on GAA repeat dynamics, we determined *FXN* transcription levels in tissues from MMR wild type mice compared with MMR knockout mice. Our results showed downregulation of *FXN* transcription associated with loss of either Pms2 or Mlh1 proteins. We also observed a similar effect in HCT-116 human cells, which are non-GAA repeat expansion cells that have loss of both MLH1 and PMS2 activities. Hence, we conclude that Mlh1 and Pms2 proteins may affect FRDA through two different mechanisms: an error prone MMR-dependent system that promotes GAA repeat expansions and a MMR-independent system that enhances *FXN* transcription.

## Materials and Methods

### Animal procedures

In this experiment YG22 *FXN* ^GAA^ transgenic mice with GAA repeat sizes ranging 90–230 repeats were utilized [18], [24]. Mice were housed in conventional open cages with Litaspen Premium 8/20 bedding, paper wool nesting and standard fun tunnel environmental enrichment. The animal husbandry was carried out at 11 h dark versus 13 h light, 20–23°C temperature and 45–60% humidity. The mice were nourished with a diet of SDS RM3 expanded food pellets and standard drinking water. All procedures were carried out in accordance with the UK Home Office ‘Animals (Scientific Procedures) Act 1986’ and with approval from the Brunel University Animal Welfare and Ethical Review Board. YG22 *FXN* ^GAA^ transgenic mice were crossed with *Pms2* or *Mlh1* heterozygous knockout mice [31], [32]. All mice were maintained in a predominant C57BL/6J (B6) genetic background. Double genetically modified mice containing the *FXN* ^GAA^ transgene with the wild type, heterozygous or homozygous MMR knockout alleles were then crossed with non-GAA transgenic mice to obtain the necessary offspring for subsequent analyses.

### Cell culture

Two human epithelial cell lines were used. NCM-460 cells, derived from normal human intestinal mucosa epithelial, were utilized as Mlh1 and PMS2 proficient cells [33], while HCT-116, derived from human intestinal carcinoma, were used as MutLα deficient cells [34]. Cells were cultured in McCoy's medium and incubated at 37°C, 5% CO_2_ and 95% humidity. Cell lines were subcultured for 10 passages.

### Trinucleotide repeat analysis

Genomic DNA was isolated from cells, tail biopsies and tissues of MMR-deficient and MMR-proficient *FXN* ^GAA^ transgenic mice by standard phenol/chloroform extraction and ethanol precipitation. PCR amplification was performed to determine the GAA repeat size of the *FXN* gene (NG_008845) using 200–500 ng of DNA, a conventional PCR kit (Qiagen), GAA-F and GAA-R primers and PCR conditions as previously described [1]. GAA PCR products were resolved in 20 cm-long, 1.5% agarose 1× TBE gels by electrophoresis at 60–90 V for 16–20 h and GAA band sizes were determined with comparison with 100 bp DNA size markers (Invitrogen). GAA repeat sizes were assessed by subtracting 451 bp of flanking non-repeat DNA from the PCR product size and dividing the remaining base pair size by 3. Following assessment of GAA repeat sizes, each individual GAA repeat of the offspring was compared with the corresponding GAA repeat of the parent, to investigate the presence/absence and direction of any intergeneration GAA repeat instability. For samples that demonstrated three GAA repeats, the transmission order of the higher, middle and lower GAA repeat from parent to offspring was considered to be maintained. A GAA repeat size in the offspring that was larger than the parent indicated an expansion, while one that was smaller indicated a contraction. Additionally, parent and offspring GAA repeats of the same size represented no change in the GAA repeat size. To determine the mutability level, the combined number of the expanded and contracted GAA repeats were divided by the total number of GAA repeats analysed.

To determine presence or absence of somatic GAA repeat instability, GAA PCR products from genomic DNA samples of several different tissues from each mouse were run on 1.5% agarose gels and qualitatively analysed. Stability was indicated by the presence of discreet GAA bands, while instability was identified as a smear of GAA PCR products, and for expansion instability there is a generalized shift to larger GAA repeat sizes.

### Mismatch repair genotype analysis

A multiplex MMR-PCR was carried out for the *Pms2* gene (NC_000071) or the *Mlh1* gene (NC_000075) on each double genetically altered mouse genomic DNA sample using 200–500 ng DNA, *Pms2*-F: ACAGTTACATTCGGTGACAG and *Pms2*-WT: ACTAATTCCCCTACGGTTTAG/*Pms2*-KO: TTTACGGAGCCCTGGCGC, or *Mlh1*-F: TGTCAATAGGCTGCCCTAGG and *Mlh1*-WT: TTTTCAGTGCAGCCTATGCTC/*Mlh1*-KO: TGGAAGGATTGGAGCTACGG primers and PCR conditions were designed as 2 minutes at 95°C, followed by 35 three-step cycles (45 sec at 95°C, 30 sec at 49°C and 45 sec at 72°C) for Pms2-PCR, or 35 three-step cycles (45 sec at 95°C, 30 sec at 55°C and 45 sec at 72°C) for Mlh1-PCR, and ultimately terminated with incubation at 72°C for 10 minutes. PCR products were resolved in 1.5% agarose 1× TBE gels by electrophoresis at 60–70 V for 20 minutes, compared with 1 Kb^+^ size marker (Invitrogen).

### Reverse transcription quantitative PCR

To determine the somatic *FXN* transcription level of double genetically-modified transgenic mice, total RNA from two FRDA-relevant tissues (brain and cerebellum) was isolated using TRIzol, following supplier guidelines (Invitrogen). The same procedure was applied to assess the *FXN* transcription level in NCM-460 and HCT-116 human cell lines. Total RNA was treated to prevent DNA contamination using DNase I (amplification grade, Invitrogen). Further to suitable RNA quality determination by *A*_260_/*A*_280_ ratio analysis by NanoDrop spectrophotometry (Invitrogen) and agarose gel electrophoresis, total RNA samples were adjusted to 500 ng/µl with DEPC water and cDNA was subsequently prepared by applying AMV reverse transcriptase (Invitrogen) and oligo-dT primers. Relative reverse transcription quantitative PCR (RT-qPCR) was performed using cDNA, power SYBR green mastermix (Applied Biosystems) and previously described *FXN* primers [23] according to the MIQE guidelines [35]. Mouse *Gapdh* was used as the reference gene in mouse somatic cells and human *GAPDH* was used as the reference gene in human cells, using previously described primers [23]. RT-qPCR reactions were performed in triplicate for each biological sample (n = 2–4) in a real-time PCR machine (ABI prism 7900HT, Applied Biosystems) and relative quantification values were identified by 2^−ΔΔCt^ method using SDS 2.4 and RQ manager software (Applied Biosystems).

### Statistical analysis

Statistical analyses were carried out using Microsoft excel 2007 software. Significant differences of the frequency distributions of the GAA repeat size intergenerational transmission (including three categories of “GAA repeat expansions”, “No change” and “GAA repeat contractions”) between groups of wild type, heterozygous or homozygous MMR knockout parental genotypes were determined by Chi squared (*x* ^2^) analysis. All other measurements, comparing two groups of sample were analyzed using student's *t* test. A *P* value of 0.05 was chosen as the significance threshold.

## Results

### Mlh1 activity promotes intergenerational GAA repeat expansions

**Figure 1:**
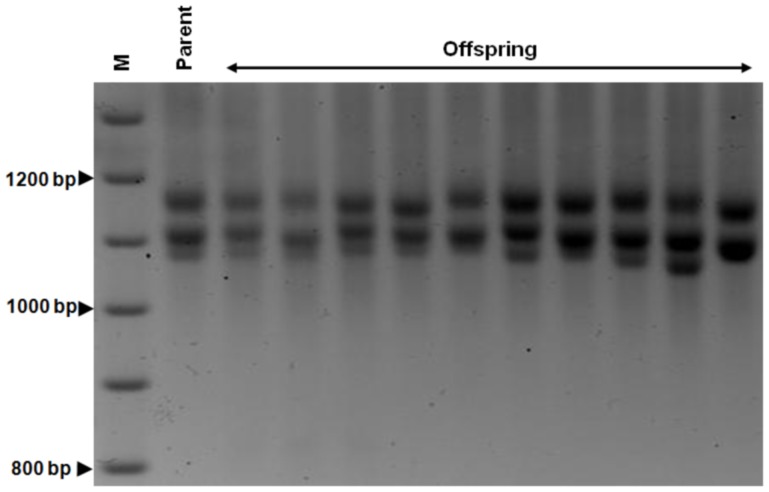
Intergenerational GAA repeats. Representative example of the ethidium bromide-stained agarose gels used to determine the GAA repeat sizes, showing GAA PCR products obtained from a YG22 GAA^+^/*Mlh1* ^+/+^ parent and 10 GAA^+^ offspring. M = 100 bp DNA size marker.

**Figure 2:**
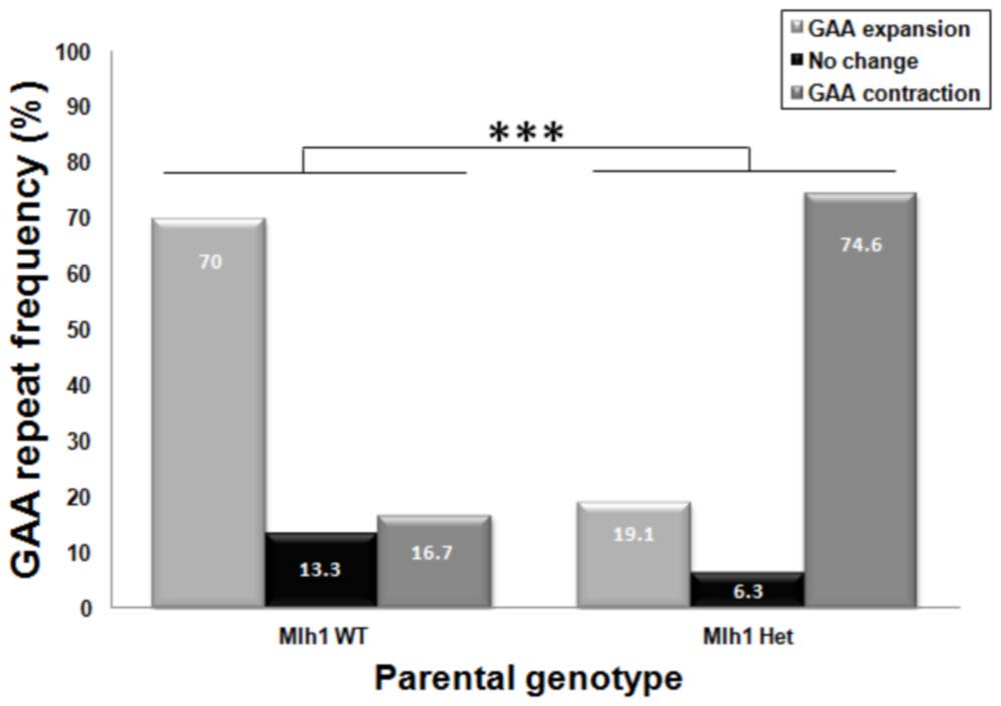
Intergenerational GAA repeat frequencies. Analysis of intergenerational transmission of GAA repeat expansion frequency based on the parental genotype (WT = GAA^+^/*Mlh1* ^+/+^, Het = GAA^+^/*Mlh1* ^+/−^). Frequencies of ‘GAA expansions’, ‘no change’ and ‘GAA repeat contractions’ transmitted to offspring are represented as percentages of total GAA repeat transmissions. Chi squared (*x* ^2^) statistical testing was applied to this expriment. WT n = 30 GAA PCR products from 10 mice; Het n = 63 GAA PCR products from 21 mice; *** = p<0.001.

We have previously assessed the effects of Pms2 protein on intergenerational transmission of the YG8 and YG22 *FXN* ^GAA^ transgenic mice by comparing GAA repeat sizes of offspring with those of parents containing one of three *Pms2* genotypes: *FXN* ^GAA^/*Pms2* ^+/+^, *FXN* ^GAA^/*Pms2* ^+/−^, *FXN* ^GAA^/*Pms2* ^−/−^ [20]. Loss of Pms2 showed an increased mutability frequency, biased towards GAA repeat expansion in an allele-dose dependent manner. In the current study, the effect of Mlh1 on intergenerational transmission of the YG22 *FXN* ^GAA^ mouse model was similarly assessed by comparing GAA repeat sizes of offspring with those of parents containing different Mlh1 genotypes: *FXN* ^GAA^/*Mlh1* ^+/+^ (parents = 2, offspring = 10) or *FXN* ^GAA^/*Mlh1* ^+/−^ (parents = 4, offspring = 21; Fig. 1). However, study of *Mlh1* ^−/−^ male and female parents was not feasible, since they are sterile due to lack of the Mlh1 protein [31]. The transmitted GAA repeat sizes were determined (see Materials and Methods) and categorized into ‘GAA repeat expansions’, ‘no change’ or ‘GAA repeat contractions’ subsets and the percentage of frequencies, as well as mean size changes of each subset, were determined to give an overall ‘transmission profile’. Analysis of data demonstrated a mutability level of 86.7% in the *FXN* ^GAA^/*Mlh1* ^+/+^ transmission profile with a bias towards GAA repeat expansions (Fig. 2). With loss of one *Mlh1* allele, the mutability level of the *FXN* ^GAA^/*Mlh1* ^+/−^ intergenerational transmission profile increased to 93.7% with a bias towards GAA repeat contractions (Fig. 2, Table 1). This suggests that down-regulation of Mlh1 protein leads to further GAA repeat instability, with a switch from expansions to contractions. Furthermore, the mean variations of GAA repeat size (i.e. the summation of total GAA repeat changes divided by the entire number of GAA repeat transmissions) for *Mlh1* ^+/−^ shows a tendency towards contraction compared with *Mlh1* ^+/+^ (Table 2). These findings suggest that Mlh1 protein can protect against intergenerational GAA repeat contractions and may also promote GAA repeat expansions.

**Table 1:** χ^2^ and *p* value analyses of intergenerational GAA repeat transmissions.

*Mlh1* parental genotype 1	*Mlh1* parental genotype 2	χ^2^ value	df	*P* value
***Mlh1*** **^+/+^**	***Mlh1*** **^+/^** ^−^	28.2	2	<0.001

**Table 2:** Mean intergenerational GAA repeat size variations.

Parental genotype	Mean GAA repeat size increase of expansions	Mean GAA repeat size decrease of contractions	Mean GAA repeat size variation of all transmission
***Mlh1^+/+^***	+3.3	−2.6	+1.86
***Mlh1*** **^+/^** ^−^	+2.3	−3.4	−2.095

### Mlh1 activity also promotes somatic GAA repeat expansions

**Figure 3:**
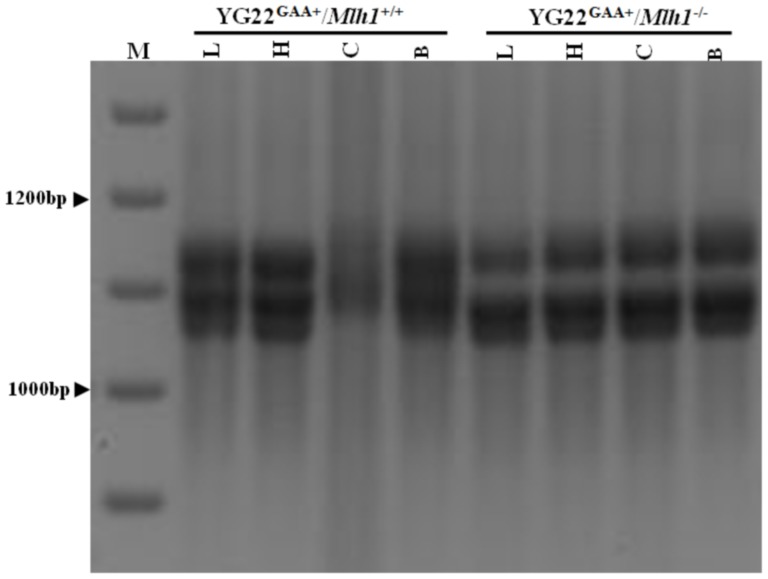
Effect of Mlh1 on somatic GAA repeat dynamics. Representative image of the ethidium bromide-stained agarose gels used to determine GAA repeat expansion dynamics from different tissues of 3–5 month-old mice in absence of presence of Mlh1. M = 100 bp size marker, B = brain, C = cerebellum, H = heart, L = liver. WT (*Mlh1^+/+^*) n = 2, KO (*Mlh* ^−/−^) n = 7.

We have previously demonstrated that loss of Pms2 leads to increased GAA repeat expansions in FRDA-relevant tissues, namely brain, cerebellum and DRG [27]. In this current study, the effect of Mlh1 on the dynamics of somatic GAA repeat size was qualitatively investigated by analyzing two neuronal tissues (brain and cerebellum) and two non-neuronal tissues (heart and liver), from either *FXN* ^GAA^/*Mlh1* ^+/+^ (Mlh1 proficient) or *FXN* ^GAA^/*Mlh1* ^−/−^ (Mlh1 deficient) 3–5 month-old mice. Analysis of somatic GAA repeats from *FXN* ^GAA^/*Mlh1* ^+/+^ mice demonstrated expansion instability in cerebellum tissue, which exhibited a smear of GAA PCR products ranging from 210 to 236 GAA repeats, compared with the other tissues, which contained discrete PCR products ranging from 201 to 230 GAA repeats; Fig. 3), consistent with our previous studies [18]. However, no such GAA repeat instability was observed in any of the somatic tissues from *FXN* ^GAA^/*Mlh1* ^−/−^ mice, which comprised of discrete PCR products ranging from 200 to 230 GAA repeats, indicating that lack of Mlh1 protein leads to GAA repeat stabilization. This finding suggests an essential role for Mlh1 in somatic GAA repeat expansion instability.

### Effects of Pms2 and Mlh1 on *FXN* transcription

**Figure 4:**
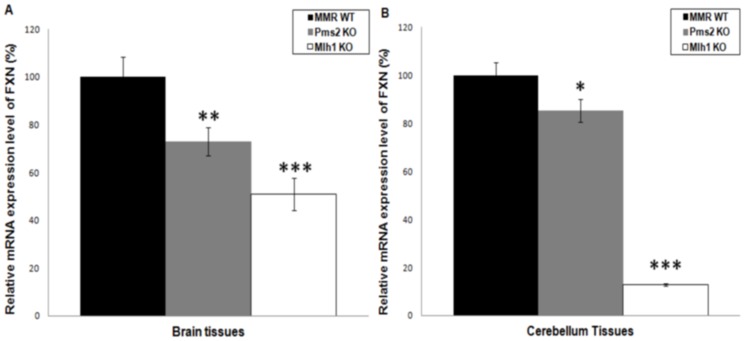
Effect of MutLα components on somatic *FXN* transcription *in vivo*. Relative RT-qPCR analyses of somatic *FXN* transcription level based on the MMR genotype (WT, *Pms2* KO or *Mlh1* KO) in (**A**) *FXN* ^GAA^/*MMR* brain tissues (n = 2–4), and (**B**) *FXN* ^GAA^/*MMR* cerebellum tissues (n = 2–4). Statistical analysis of the experiment was perform using the student's *t* test. Error bars = S.E.M, * = p<0.05, ** = p<0.01, *** = p<0.001.

Previous studies have detected an inverse correlation between GAA repeat expansion size and *FXN* transcription level in FRDA [23], [36]. To determine any effect of Pms2 or Mlh1 deficiency on *FXN* transcription, in addition to their effects on somatic GAA repeat instability, we investigated brain and cerebellum tissues from YG22 transgenic mice. In each case, two subgroups were employed based on the MMR genotypes: *FXN* ^GAA^/*MMR* ^+/+^ and *FXN* ^GAA^/*MMR* ^−/−^. Relative quantification of *FXN* transcription in brain tissues identified a decline of approximately 30% for *FXN* ^GAA^/*Pms2* ^−/−^ transgenic mice compared to *FXN* ^GAA^/*Pms2* ^+/+^ mice (Fig. 4A). Similarly, cerebellum tissues revealed a decrease of *FXN* transcription in Pms2-deficient transgenic mice compared with Pms2-proficient mice (Fig. 4B). These findings demonstrate that lack of Pms2 leads to reduced levels of *FXN* transcription in FRDA-relevant tissues, possibly due to the increased sizes of GAA repeats. Further analysis of brain tissues showed that deficiency of Mlh1 led to downregulation of *FXN* transcription to approximately 50% of the levels in Mlh1-proficient mice (Fig. 4A). Moreover, study of cerebellum tissues revealed a dramatic decrease in *FXN* transcription in Mlh1-deficient mice to levels of approximately 10% compared with Mlh1-proficient mice (Fig. 4B). These data suggest a more crucial role of Mlh1 on regulating *FXN* transcription levels in brain and cerebellum tissues compared with Pms2.

### MutLα dysfunction reduces *FXN* transcription in human cells

**Figure 5:**
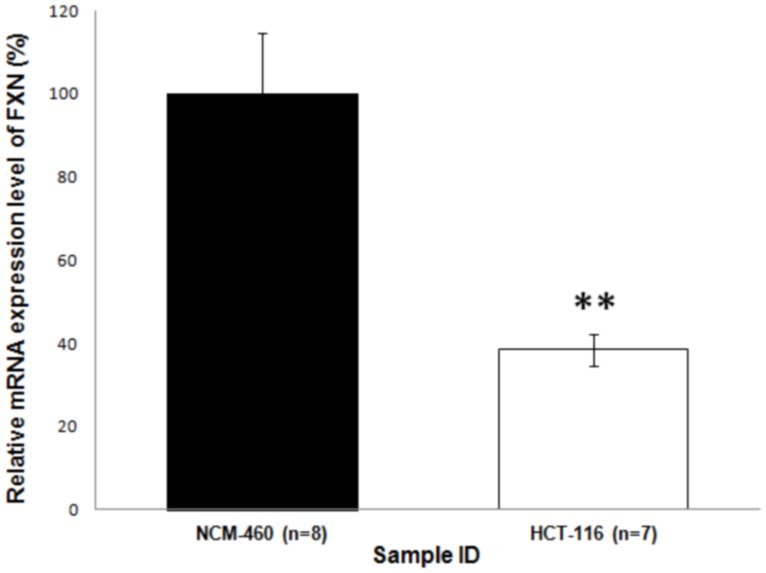
Effect of the MutLα-complex on *FXN* transcription *in vitro*. Relative RT-qPCR analyses of the mean *FXN* transcriptional level, isolated from MMR-proficient human cells, NCM-460, and MutLα heterodimer-deficient cells, HCT-116 (n = 3). Statistical analysis of the experiment was perform using the student's *t* test. Error bars = S.E.M, * = p<0.05, ** = p<0.01, *** = p<0.001.

To investigate if MutLα-heterodimer proteins also have an effect on *FXN* transcription in human epithelial cell lines, we compared NCM-460 (human MMR-proficient) cells with HCT-116 (human MMR-deficient) cells. Observations have previously demonstrated that HCT-116 carries a mutation within the *MLH1* gene that prevents MLH1 and PMS2 protein binding, subsequently leading to dysfunction of MutLα-heterodimer complex and degradation of free PMS2 [34], [37], [38]. Comparing these cell lines revealed significantly higher *FXN* transcription in the MMR-proficient NCM-460 control cells compared with HCT-116 cell lines (Fig. 5). This demonstrates a consistent effect of MLH1/PMS2 deficiency on *FXN* transcription in both human cells *in vitro* and *FXN* ^GAA^ mouse tissues *in vivo*. Overall, these findings suggest an essential role for MutLα-heterodimer proteins in the regulation of *FXN* transcription.

## Discussion

### MutLα proteins play distinct roles in both intergenerational and somatic GAA repeat instability in FRDA

We have previously demonstrated an important role for Pms2 protein in promoting GAA repeat contractions and protecting GAA repeats against further expansions in transmission from parents to offspring [20]. It has also been demonstrated that Pms2 inhibits somatic GAA repeat expansions in neuronal tissues [27]. To further investigate the role of the MMR system in FRDA, we analyzed the effect of Mlh1 protein on intergenerational and somatic GAA repeat expansion instability in YG22 *FXN* ^GAA^ transgenic mice. With loss of Mlh1, intergenerational GAA repeat mutability increases, agreeing with the accepted role of Mlh1 within the MutLα-heterodimer complex of the MMR system, to interact with one of MutSα (Msh2-Msh6) or MutSβ (Msh2-Msh3) heterodimers and continue the DNA repair process during replication and recombination [29], [39]. However, further analysis of Mlh1 dysfunction showed that the increased mutability of the expanded GAA repeats was biased towards contractions and hence activity of this protein actively promotes GAA repeat expansions. This is not compatible with the canonical DNA repair function of Mlh1 within the MMR system. It is not yet clear how Mlh1 activity is able to promote intergenerational transmission of GAA repeat expansions. One possibility is that the MMR system promotes expansions in GAA repeat regions through an unknown error-prone mechanism.

**Figure 6:**
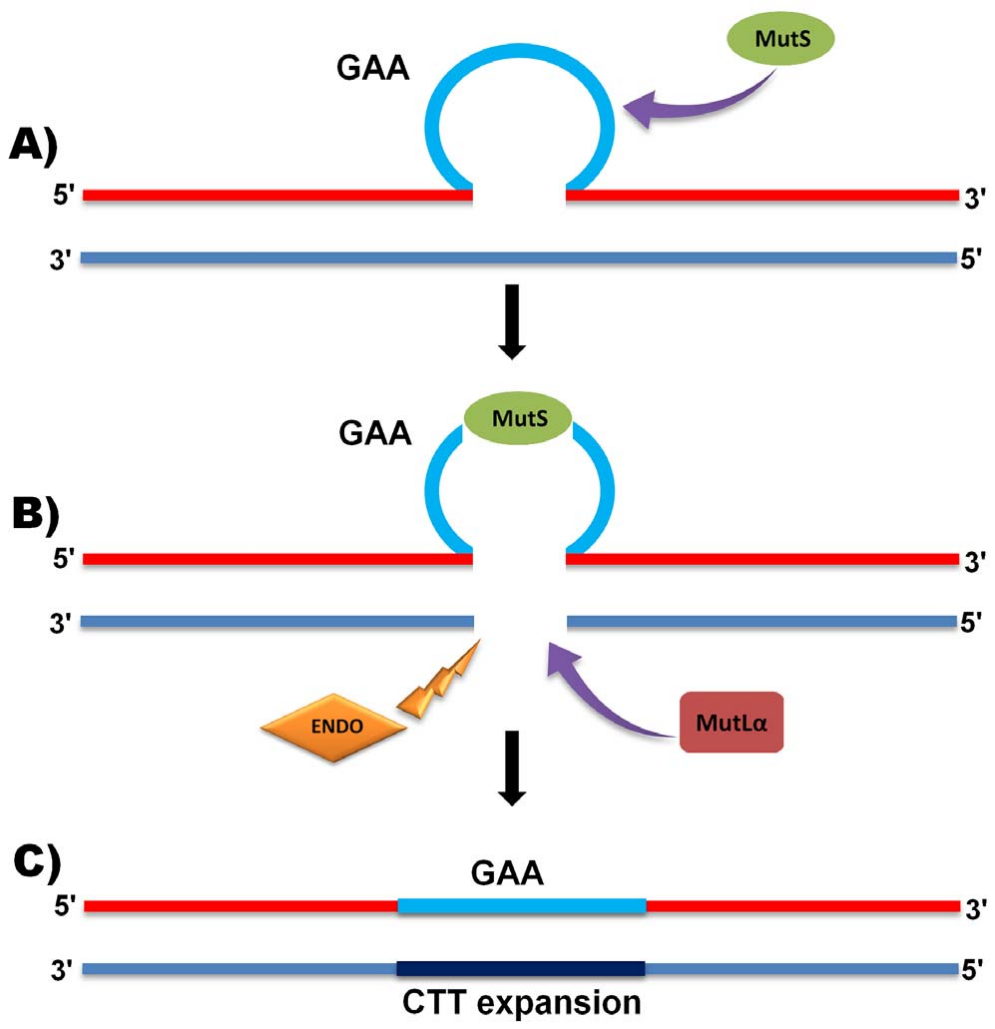
Proposed mechanism of MMR action on intergenetional GAA repeat expansions. Schematic images representing: (**A**) a small loop caused by triplex DNA structure; (**B**) recognition of the loop by the MutS complex, cleavage with endonuclease (ENDO), opening of the loop, recruitment of MutLα and synthesis of expanded DNA, and (**C**) end of repair by ligation of the further expanded strand and release of MMR proteins.

It is generally accepted that repeat expansions can form different types of non-B DNA structures during DNA replication. Meanwhile, it has been demonstrated that GAA repeat expansions can also form triplexes between intramolecular GAA**•**GAA**•**TCC (R**•**R **•**Y) sequences [40], or even more complicated form of sticky DNA by binding two separate GAA repeats in naked supercoiled DNA [41], [42]. Similar sequences might lead to incompetent DNA replication. Evidence indicates that the MMR system may be recruited to correct such non-canonical DNA structures [43], although the mechanism might be different for intergenerational GAA repeat instability compared with somatic GAA repeat instability. Briefly, in the case of intergenerational GAA repeat expansion, a small loop which is produced as a consequence of GAA triplex formation could initially be recognized by MutSα or MutSβ complex (Fig. 6A). Interestingly, it has previously been demonstrated that occupancy of MutSβ protein components, Msh2 and Msh3, and to a lesser extent Msh6, is increased in the downstream region of expanded GAA repeat sequences, presumably due to binding to the expanded GAA repeats [20], [44]. These data suggest a higher likelihood of the MutSβ complex to bind to the GAA repeat region, although further studies are required to confirm this. In any event, one of the MutS complexes may initiate the MMR mechanism by recognizing and binding to a DNA triplex loop of expanded GAA repeat sequences [43], [45]. Following MutS complex binding to the triplex DNA loop, a MMR-directed excision might be developed by endonuclease activity [45] within the single-strand of DNA facing opposite to the loop. This event may subsequently lead to recruitment of the MutLα complex and other coordinating factors to proceed with the MMR system (Fig. 6B) [45]. Concurrently, an alternative enzyme (presumably a helicase [46]) may open up the triplex sequence, creating a gap in the nicked opposing strand of DNA, which is filled with additional sequence by a DNA polymerase (Fig. 6C) [28], [47]. Thus, this event may not only resolve the GAA repeat triplex structure [45], but may at the same time cause further GAA repeat expansions. However, this proposed mechanism does not account for how loss of Pms2 can cause the observed increased magnitude of GAA repeat expansions. Hence, we would suggest that Pms2 can promote GAA repeat contractions by another mechanism. Moreover, this mechanism is only able to explain the potential correlations of GAA repeat expansions, DNA triplex structure formation and MMR-error prone activity in the cells with DNA replication ability (e.g. mitotic cells, such as germ cells or stem cells), hence it is not able to demonstrate the mechanism whereby MMR activity promotes GAA repeat expansions in non-dividing adult somatic cells (i.e. post mitotic cells, such as mature neuronal cells).

**Figure 7:**
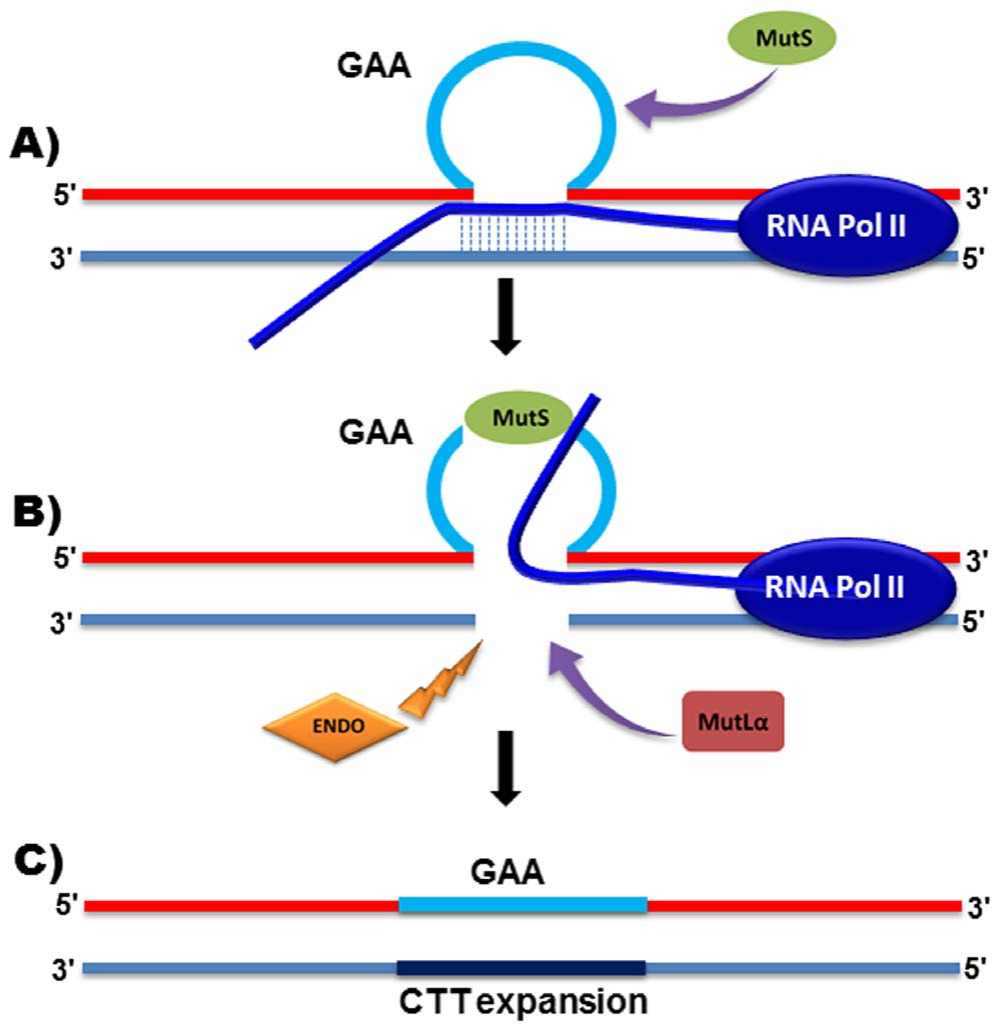
Proposed mechanism of MMR action on post-mitotic somatic GAA repeat expansions. Images represent: (**A**) a small GAA loop is formed as part of the triplex DNA**•**RNA R-loop structure caused by transcription within GAA repeats; (**B**) recognition of the small GAA loop by MutS-heterodimers, cleavage of the CTT DNA strand with an endonuclease (ENDO) and recruitment of MutLα, and (**C**) release of the RNA, synthesis of expanded DNA and end of repair by ligation of the expanded strand and release of MMR proteins.

The results of our somatic GAA repeat expansion studies showed that loss of Mlh1 protein leads to stabilization of the expanded GAA repeats in somatic tissues, either due to protection against further GAA repeat expansions or promotion of GAA repeat contractions in neuronal tissues. Despite detecting similar effects of Mlh1 on the dynamics of GAA repeat expansions, we would propose that the Mlh1 mechanism of action may be different between intergenerational transmission and somatic cells. Previous studies have suggested that binding of premature GAA repeat mRNA with the relevant DNA sequence can produce an unusual DNA**•**RNA triplex (R-loop) structure *in vitro* and in bacteria [4], [48], which culminates in stalling of RNA polymerase II [49], [50]. Therefore, in the case of post mitotic GAA repeat expansions, a non-canonical R-loop structure might be created during binding of single-stranded template TTC repeat sequences to transcribed GAA mRNA, forming small loop-outs of the single stranded non-template GAA repeats (Fig. 7A) [4], [48]. Evidence shows that these small loop-outs may then be recognized by one of the MutS complexes, subsequently binding with the MutLα complex (Fig. 7B) [51], [52]. As part of the repair mechanism, a MMR-directed excision may be made in the TTC template strand of DNA (Fig. 7B) [28] and the stalled mRNA may be released from the template strand by helicase enzyme activity. Subsequently, the MutS and MutLα complexes may recruit other MMR system factors (i.e. PCNA, RFC and DNA polymerase [45]) to repair the cut TTC strand of DNA, but with the introduction of expanded repeat sequences (Fig. 7C). Since GAA repeat expansions occur predominantly in tissues that contain mainly non-dividing cells (e.g. brain and cerebellum), a transcriptional-based mechanism may indeed be the most pertinent model to explain the role of the MMR system in somatic GAA repeat expansions.

### MutLα proteins contribute to the regulation of *FXN* transcription

**Figure 8:**
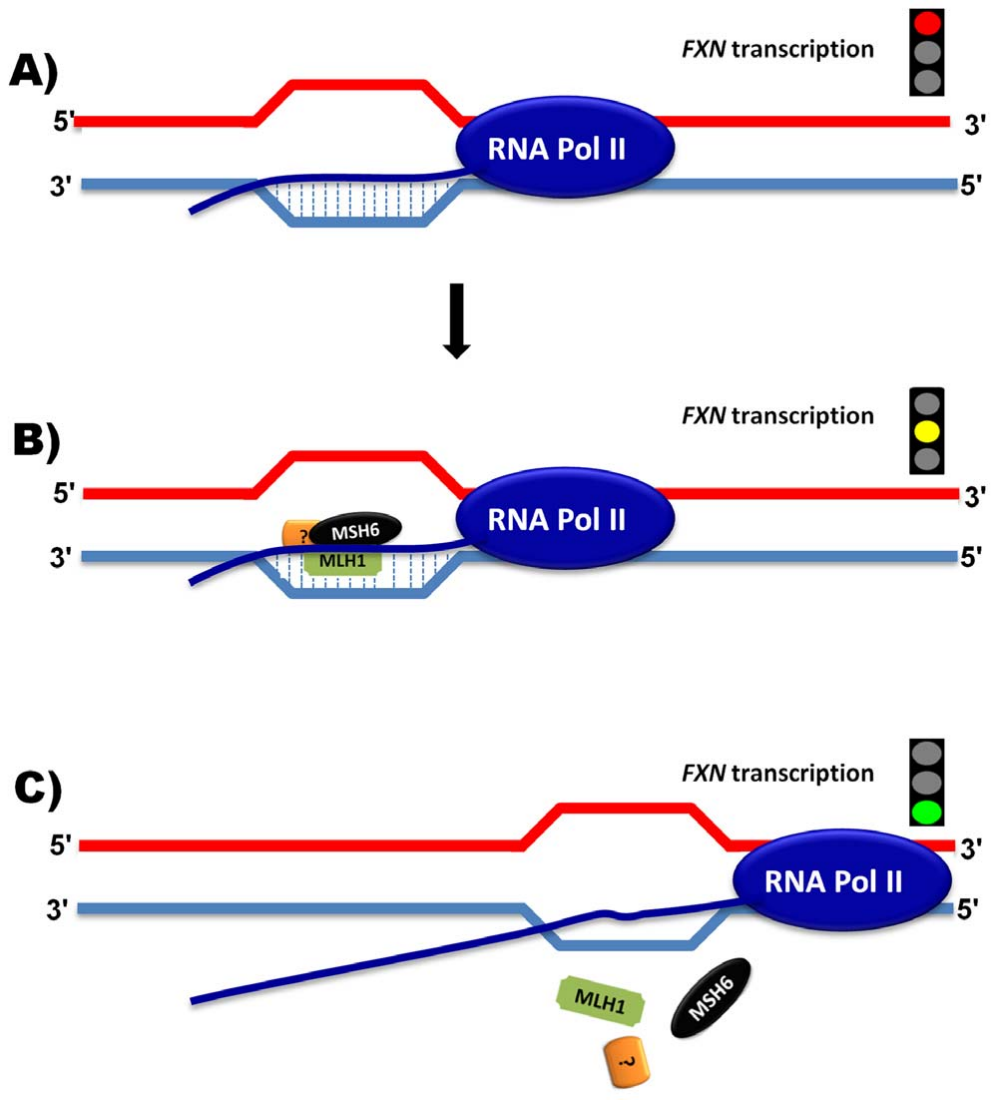
Proposed mechanism of MLH1 action on *FXN* transcription. Images illustrate: (**A**) inhibition of transcription by triplex DNA**•**RNA R-loop formation; (**B**) binding of MLH1 and MSH6 to the R-loop, together with other unknown factors (?), and (**C**) release of bound premature mRNA to allow continuation of transcription.

In this study, we also identified an effect of MutLα-heterodimer proteins on *FXN* transcription. Thus, disruption of either Mlh1 or Pms2 was shown to downregulate *FXN* transcription *in vivo* and *in vitro*. Curiously, this mode of action is associated both with and without somatic GAA repeat instability (Table 3). Thus, loss of Pms2 causes further increased somatic GAA repeat expansions and a corresponding down-regulation of *FXN* transcription. In contrast, Mlh1 disruption also results in down-regulation of *FXN* transcription, in spite of a corresponding loss of somatic GAA repeat expansion (Table 3). Although the reasons for these conflicting results are not yet clear, it is proposed that another mechanism, distinct from the MMR system, which is not involved in GAA repeat expansions, might lead to down-regulation of *FXN* transcription with Mlh1 deficiency. Interestingly, in addition to its contribution to the MMR system, the MLH1 protein is also able to cooperate with the transcriptional-coupled nucleotide excision repair (TC-NER) system to amend genomic errors with bulky helix loops [53], [54]. The TC-NER system is generally recruited to repair genomic errors, led by preferentially binding transcription sequences and RNA polymerase II to the transcribed DNA strand in the expressed genes [54], [55]. Investigations of HCT-116 *MLH1*-deficient cells have revealed hypersensitivity to UV light due to dysfunction of TC-NER, while artificially introduction of *MLH1* gene to this cell line leads to TC-NER system activity and hence reduced sensitivity to UV light [54], [56]. It has also been reported that dysfunction of TC-NER system down-regulates transcription of particular genes [57]. These observations suggest that there may be a similar MLH1 mechanism of action on the regulation of *FXN* transcription. In our proposed model, a complex of unwound DNA, primary *FXN* mRNA sequence and RNA polymerase II may combine to form an unusual R-loop structure (Fig. 8A), leading to further somatic GAA repeat expansions on the one hand and *FXN* transcription blockage on the other. MLH1 may act in a complex with other proteins (e.g. MSH6) in the TC-NER system to repair the R-loop errors and consequently increase *FXN* transcription (Fig. 8B). Activation of this protein complex might assist in the release of stalled RNA polymerase II, culminating in resumption of *FXN* transcription (Fig. 8C). In contrast, without MLH1, the TC-NER protein complex would be incomplete and non-functional. Therefore, RNA polymerase II would remain stalled due to failure of the TC-NER system (Fig. 8A) and blockage of *FXN* transcription would persist.

**Table 3:** Effect of MutLα-heterodimers on GAA repeat expansion dynamics and *FXN* transcription levels.

	Intergenerational GAA repeat expansions	Somatic GAA repeat expansions	Brain and cerebellum *FXN* transcription level
**Pms2 deficiency**	Increased	Increased	Decreased
**Mlh1 deficiency**	Decreased	Decreased	Decreased

### Conclusions

Our findings highlight the importance of the MutLα components, PMS2 and MLH1, in both the dynamics of GAA repeat expansion and *FXN* transcription. We conclude that, in addition to previously proposed therapeutic option to inhibit MSH3 action [20], identifying compounds which upregulate PMS2 activity could perhaps be considered as a therapeutic approach to prevent progressive GAA repeat expansion instability and elevate *FXN* expression. However, great caution should be taken since previous studies have reported hypermutability, DNA damage tolerance and tumorigenesis as consequence of PMS2 overexpression [58]. In addition, compounds that upregulate MLH1 activity and hence cause increased *FXN* expression could also be considered as a potential FRDA therapy. However, in this case GAA repeat expansions might simultaneously be promoted in FRDA cells with an independent mechanism of action to *FXN* transcription. Thus, further investigations will be required to clarify the precise mechanisms of action of MLH1 in GAA repeat instability and *FXN* transcription before being fully considered as a target for FRDA therapy.
